# A simple, efficient, and safe way of finding recurrent laryngeal nerve beneficial for PTC patients

**DOI:** 10.1097/MD.0000000000020138

**Published:** 2020-05-08

**Authors:** Shouyi Yan, Chao Xie, Wenxin Zhao, Bo Wang, Liyong Zhang

**Affiliations:** a Department of Thyroid and Vascular Surgery; b Department of General Surgery; c Minimal Invasive Center, Fujian Medical University Union Hospital; d Fujian Medical University, Fuzhou, Fujian Province, China.

**Keywords:** improved method, papillary thyroid cancer, parathyroid grand, recurrent laryngeal nerve, thymus-related inferior parathyroid gland

## Abstract

**Background:**

More surgeons have known the importance of parathyroid grand and recurrent laryngeal nerve protection in the surgery, but there is still plenty of scope to improve the surgical techniques. This study aims at investigating whether the improved method of finding recurrent laryngeal nerve (RLN) can protect parathyroid grand and RLN.

**Methods:**

One hundred fifty-eight patients were enrolled and divided randomly into the test and control group according to different methods of finding RLN in the surgery. In the experimental group the author could quickly find the laryngeal recurrent nerve in the lower part of the neck and separate along the surface of the recurrent laryngeal nerve to the point where the recurrent laryngeal nerve gets into the larynx close to the thyroid gland named lateral approach, while in the control group the author severed the middle and lower thyroid vein and raised the lower thyroid pole to look for the RLN near the trachea by the blunt separation.

**Results:**

The author identified 152 and 159 parathyroid glands in the test and control group, respectively and there were a lower ratio of auto-transplantation and less operative time in the test group compared with that in the control group. The author also found that the parathyroid hormone level (1 day and 2 months) in the test group was higher than that in the control group. There were no differences in metastatic LN and recurrent laryngeal nerve palsy in the 2 groups.

**Conclusion:**

The improved method of finding RLN is a simple, efficient and safe way, and easy to implement.

## 1 Introduction

Thyroid carcinoma (TC) is the most common endocrine malignancy, and the incidence rates of TC have increased over the past 30 years.^[1]^ In 2012, 298,000 new thyroid cancer cases and 40,000 thyroid cancer deaths were supposed to occur all over the world. The percentage of the new TC cases and the TC deaths in China accounted for 15.6% and 13.8% of the world, respectively.^[2–4]^ Now the main treatment for thyroid cancer is surgery, of which the key point is to protect the recurrent laryngeal nerve (RLN) and parathyroid gland (PG). Finding nerves has become easier with the help of intraoperative neuromonitoring technique,^[5]^ but there are still some difficulties in some areas without the monitoring equipment or lack of surgical experience. In this study, some improvements were achieved in finding the RLN to investigate whether it was helpful for thyroid surgery in the protection of the RLN and PG.

## 2 Material and methods

### 2.1 Patients characteristics

A total of 158 consecutive patients were enrolled into this study from August 2018 to February 2019, and the patients’ characteristics were showed in Table 1. All the patients were diagnosed as unilateral papillary thyroid carcinoma (PTC) by preoperative fine needle aspiration. According to the methods of finding RLN, the patients were randomly divided into 2 groups: the test group (n = 76) and the control group (n = 82). The inclusion criterions were as follows:

(1) The longest PTC diameter was less than 4 cm,
(2) The patients were confirmed as unilateral PTC.

**Table 1:**
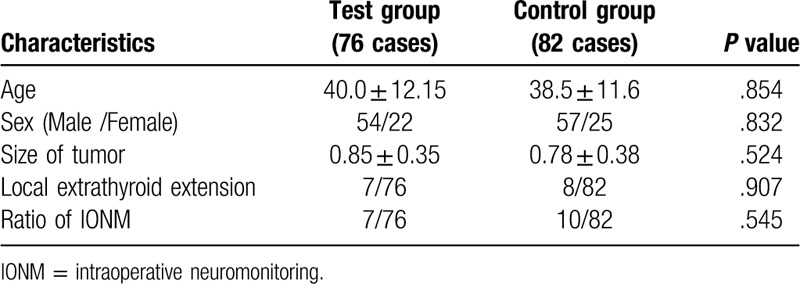
The clinical characteristics of the patients.

And the exclusion criteria were as below:

(1) Postoperative pathology suggests that there were other types of tumors, such as medullary and undifferentiated cancer,
(2) Tumor had invaded PGs during the operation,
(3) The patient had a history of thyroid surgery,
(4) The patient was less than 16 years old,
(5) The patient was unable to comply with the follow-up.

The randomization was carried out by using computer-generated random number tables. All the operations were performed by the same surgeon, and all the patients underwent thyroid lobectomy plus dissection of the central lymph node. The study was approved by the ethics committee of Fujian Medical University Union Hospital, and all patients provided an informed consent.

### 2.2 Surgical procedure of the thyroid lobe dissection

#### 2.2.1 Finding RLN in the control group

**Figure 1:**
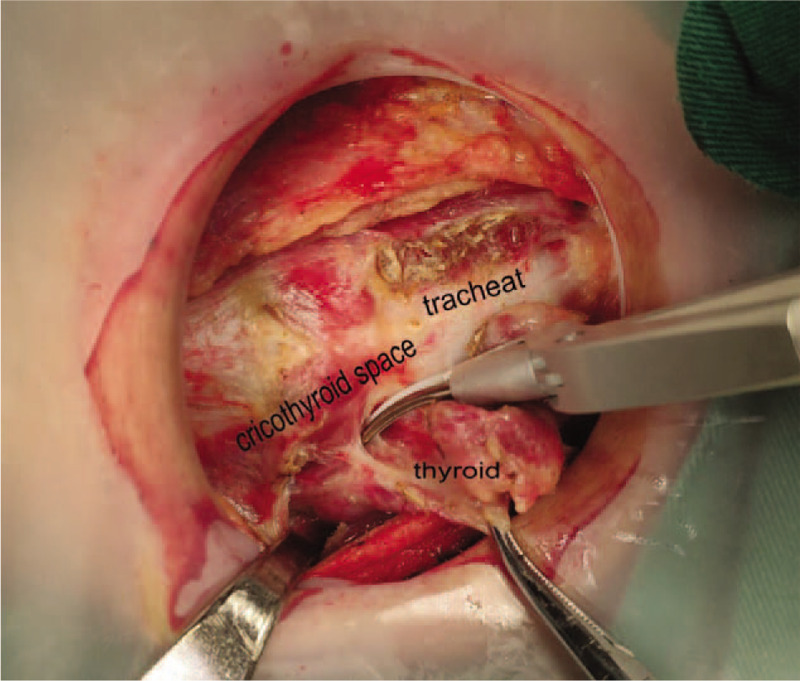
Severed the anterior branch of the superior thyroid artery along the cricothyroid space.

**Figure 2:**
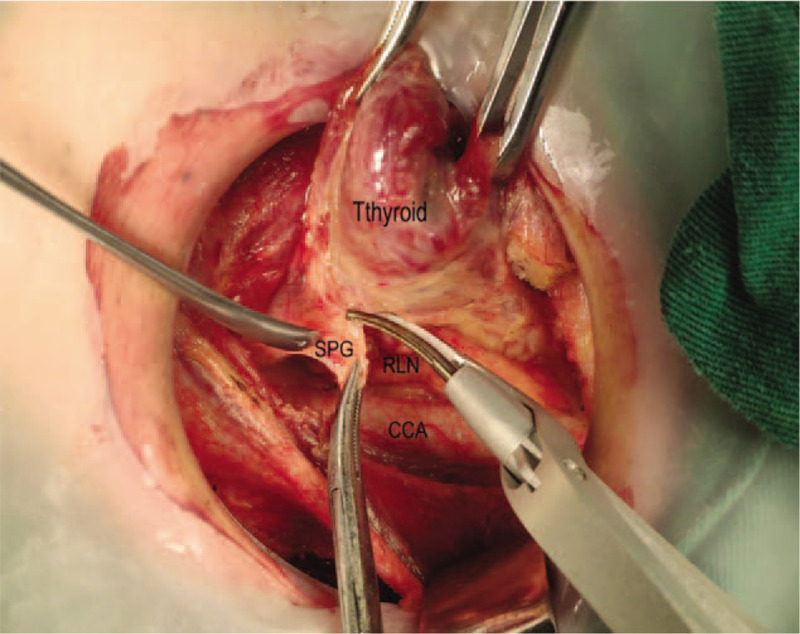
Separate upward to protect the upper parathyroid gland.

**Figure 3:**
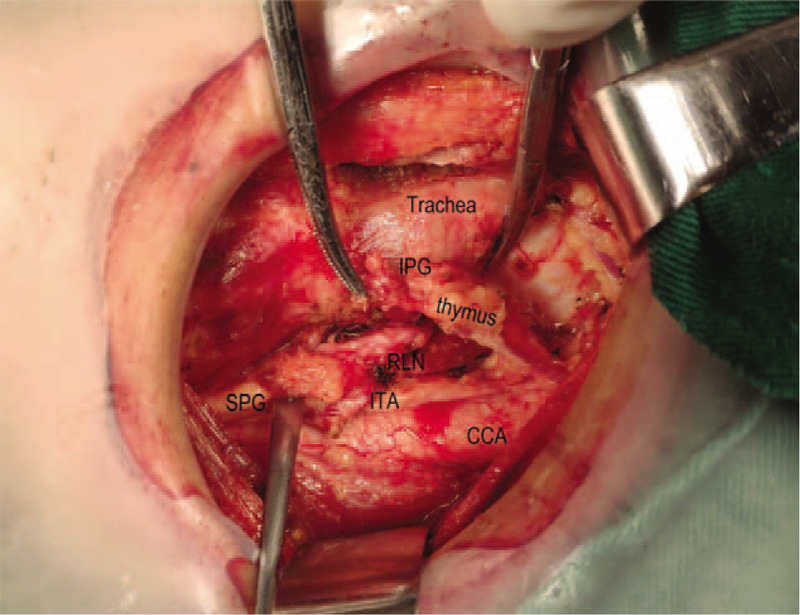
Dissection of the central lymph node was performed according to the NNCN guideline.

Step1: we severed the thyroid isthmus, and then severed the anterior branch of the superior thyroid artery along the cricothyroid space (see Fig. 1).
Step2: we severed the middle and lower thyroid vein and raised the lower thyroid pole to look for the RLN near the trachea by the blunt separation. After finding the RLN, we continued to separate along the RLN to the entry point and gradually severed the medial and lateral tissues near the RLN.
Step3: we continued to separate upward to protect the upper PG, and also assured that the whole operation was close to the thyroid gland (see Fig. 2).
Step4: all the patients were diagnosed with thyroid cancer preoperatively, so the dissection of the central lymph node was performed according to the NCCN guideline (see Fig. 3).

#### 2.2.2 Finding RLN in the test group (the lateral approach)

**Figure 4:**
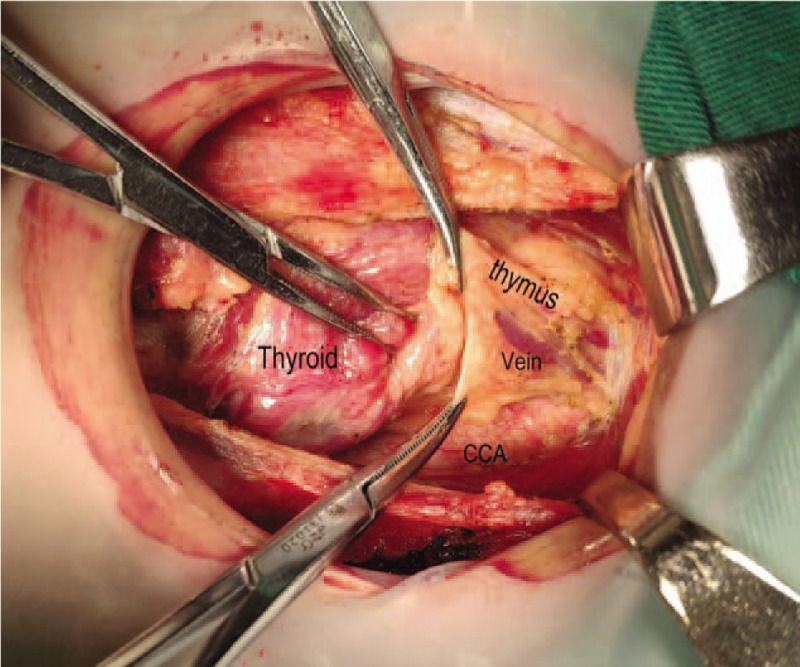
Separating downward to the thymus level.

**Figure 5:**
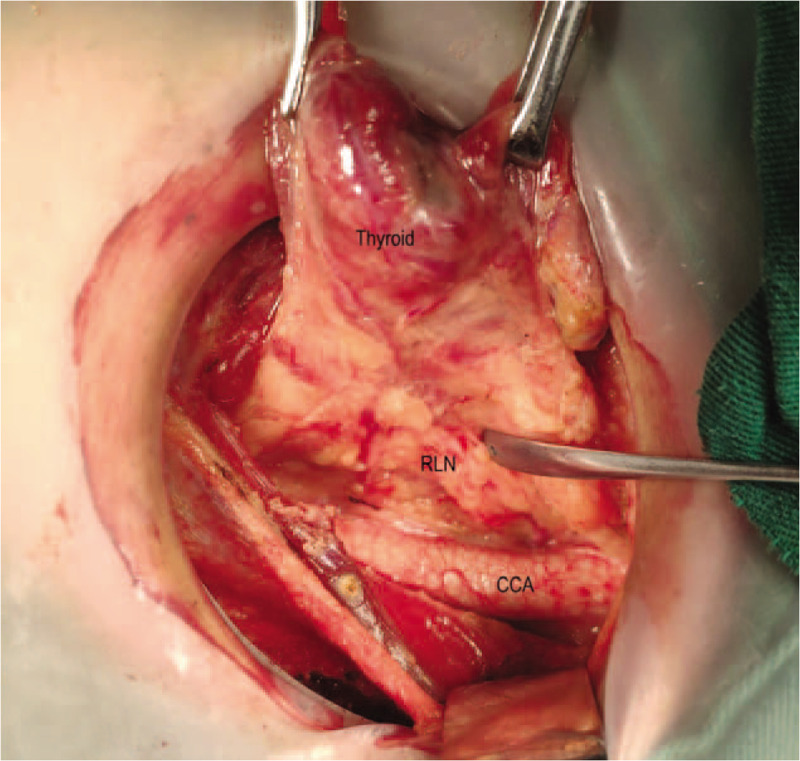
Finds the laryngeal recurrent nerve in the lower part of the neck.

**Figure 6:**
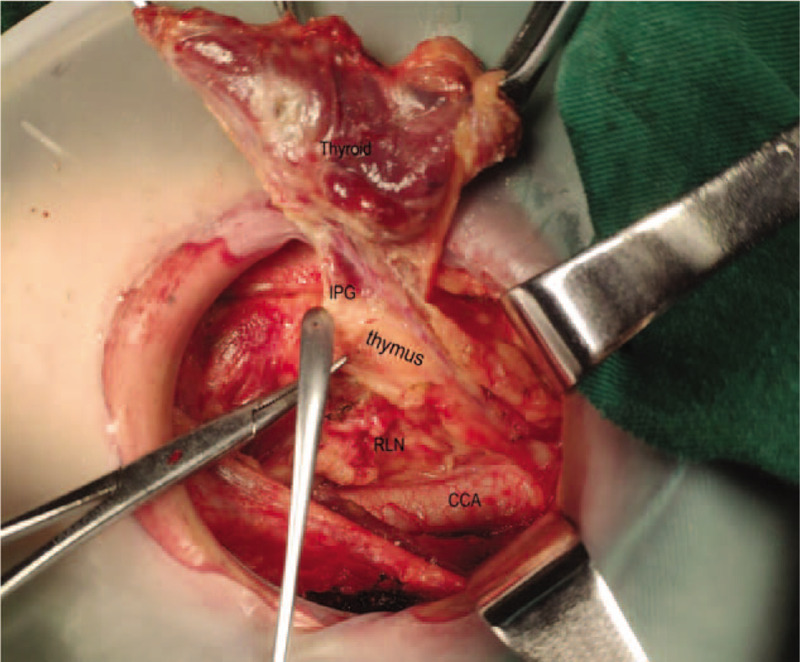
Finds the inferior parathyroid gland which was closely related to the thymus gland.

**Figure 7:**
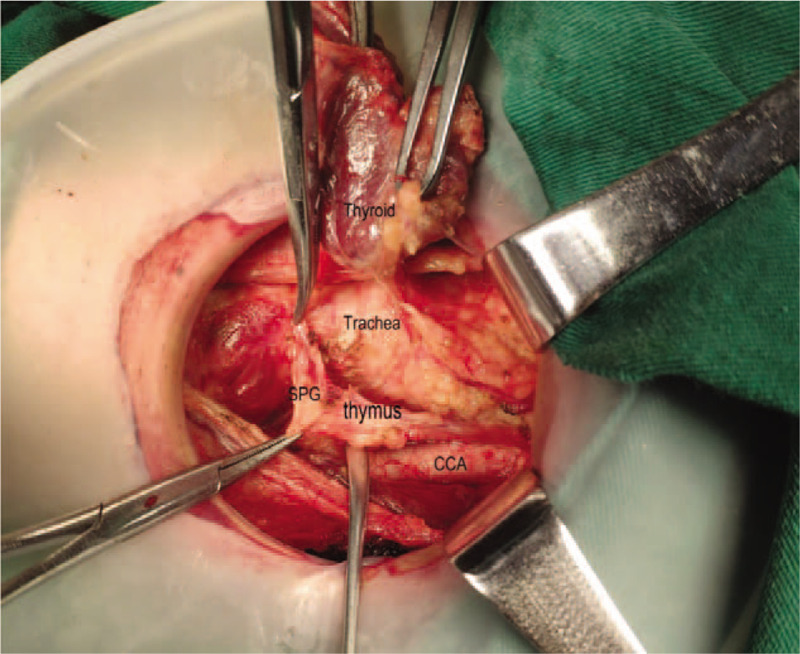
Attempt to preserve inferior parathyroid gland in situ named as thymus-related inferior parathyroid gland.

Note: CCA common carotid artery

Step 1, step 3, and step 4 are the same in the both groups. This paper focuses on step 2 with a detailed statement. First, we severed the middle thyroid vein to expose the common carotid artery, and then continued separating downward to the thymus level in the caudal direction and meanwhile assured to protect the thymus vein (see Fig. 4). The improved method mainly relies on the characteristics that the right recurrent laryngeal nerve intersects with the common carotid artery on the right side and the laryngeal recurrent nerve is close to the surface of the esophagus on the left side. Therefore, we could quickly find the recurrent laryngeal nerve in the lower part of the neck (see Fig. 5) and separate along the surface of the recurrent laryngeal nerve to the point where the recurrent laryngeal nerve gets into the larynx close to the thyroid gland. We could also find the inferior parathyroid gland (IPG) which was closely related to the thymus gland (see Fig. 6) named as thymus-related inferior parathyroid gland (TRIPG), and attempt to preserve it in situ (see Fig. 7).

### 2.3 Identification and protection of PG during the operation

It is known that the anatomic location of the superior PG is constant on the dorsal aspect of the upper thyroid lobes at the level of the inferior border of the cricoid cartilage, but the location of inferior PG is variable due to its embryologic relationship to the thymus. The key point of PG identifying is to observe the blood supply (micro vessel on the surface of PG), the texture (the hardness is between the fat and the lymph nodes), the color (light brown) and the neighboring relationship (usually connected with the thymus).^[6]^ We should try to preserve the PG in situ when looking for it in the operation (see picture 7). If the PG was lack of blood supply or completely free with thymus, it should be transplanted into the forearm in time. Also if the PG failed to be found during the operation, we should identify it in the resected specimens according to its physical characteristics, which could be confirmed by the parathyroid hormone (PTH) test paper and transplanted into the forearm timely.^[7,8]^

### 2.4 Follow-up and postoperative treatment of hypoparathyroidism

The level of PTHs would be tested in two months after the operation. Postoperative hypoparathyroidism was defined as that the level of PTHs was less than 1.3 mmol/L after 6 months. The follow-up time was 2 to 6 months for the patients. Calcium supplementation was not routinely administered to the patients, but calcium and vitamin D were routinely prescribed to the patients with symptomatic hypoparathyroidism until the level of PTH recovered to normal. Intravenous substitution of calcium was not a routine unless serious symptomatic hypocalcemia appeared. Levothyroxine treatment was necessary for all patients after the operation.

### 2.5 Data collection

General characteristics, intraoperative factors, pathologic examination, the number of lymph nodes and metastatic lymph nodes in the resected specimens, and postoperative complications were collected retrospectively. PG of accidental removal was defined as not finding the PG during the operation (including careful inspection of the resected specimens) but finding it in the final pathological examination. The seventh edition of the American Joint Committee on Cancer (AJCC) staging was used for all the recruited patients. The primary endpoints were the operative time, the numbers of the total LN and the metastatic LN, the ratio of PG auto-transplantation, the PTH level, the postoperative complications, the hemorrhage volume, the ratio of intraoperative neuromonitoring (IONM), and the postoperative hospitalization days.

### 2.6 Statistical analysis

Statistical analysis was performed by SPSS 17.0, Chicago, IL. All values are presented as mean ± standard deviation. A *T* test or Chi-Square test was used to determine statistical significance, requiring *P* < .05 was considered to be statistically significant.

## 3 Results

### 3.1 Patients characteristics (see Table 1)

The clinical characteristics of the patients in the two groups were summarized in Table 1. PTC was confirmed by the postoperative pathology for all the patients. There were no significant differences between the 2 groups in terms of age (*P* = .854), sex (*P* = .832), size of tumor (*P* = .524), extrathyroid extension (*P* = .907), and ratio of IONM (*P* = .545).

### 3.2 The identifying and protecting of PG (thymus-related PG) (see Table 2)

We found most of PG concluding conservation in situ and transplantation in the arm, but still missed some inferior PG in the control group (5/82). Pathological results revealed that 7 PG of accidental removal occurred in the control group, whereas 2 PG occurred in the test group. The difference was not statistically significant (*P* = .110).

**Table 2:**
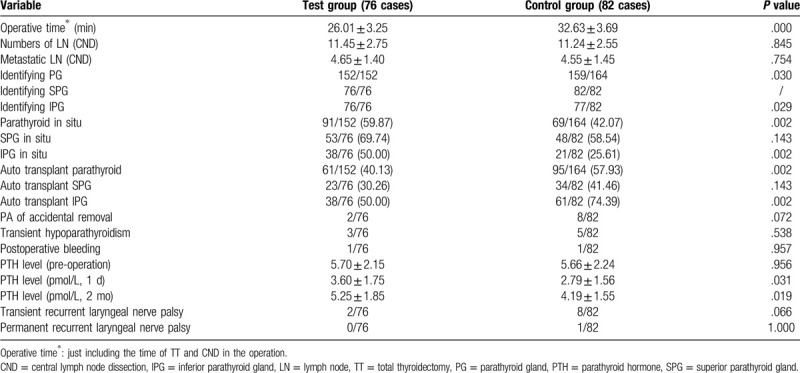
Comparison of variable in the 2 groups.

We identified 152 and 159 PG in the test group and the control group respectively, and there was a significant difference between the 2 groups (*P* = .03). Meanwhile there was a lower ratio of auto-transplantation in the test group compared with that in the control group (40.13% vs 57.93%), which was also a major difference (*P* = .003). It is also found that the PTH level (1 day) in the test group was higher than that in the control group (3.60 ± 1.75 vs 2.79 ± 1.56) with an obvious difference (*P* = .019) in the comparison of PTH level (2 month) (5.25 ± 1.85 vs 4.19 ± 1.55).

### 3.3 Central lymph node dissection in the 2 groups (See Table 2)

All the patients underwent dissection of the central lymph node, but there was no statistically significant difference between the 2 groups in the aspect of total LN and metastasis LN (*P* = .845, *P* = .754).

### 3.4 Side effects and operative complications (See Table 2)

In this study a few patients encountered the complications including postoperative bleeding (2/158), transient hypoparathyroidism (8/158), transient recurrent laryngeal nerve palsy (10/158) and permanent recurrent laryngeal nerve palsy (1/158) with no statistical difference in the 2 groups (*P* < .05). The patients of transient hypoparathyroidism did not show the low-calcium symptoms with treatment and the PTH level recovered to normal after 2 weeks. Also there was only one patient with vocal cord dyskinesia according to electronic laryngoscope after two months.

## 4 Discussion

Thyroid lobectomy plus central lymph node dissection (CND) was a premise for treating patients with PTC.^[9,10]^ An increasing number of surgeons were paying more attention to the protection of PG and RLN so as to prevent permanent hypoparathyroidism and nerve palsy which were the most common complications.^[11,12]^ It is well known that a rapid identification is the precondition for PG and RLN protection during the operation, while the identification was affected by various factors in the previous surgical methods such as obesity, fatty degeneration, bleeding.^[13,14]^ Now finding RLN is no longer a big problem, especially for the experienced surgeons and with the help of IONM.^[15,16]^ In the previous surgical methods, we usually isolated the inferior thyroid vessels and exposed the RLN with blunt dissection beside the trachea. If hemorrhage, larger grand and tight adhesion occurred in the operation mentioned above, it was prone to cause RLN injury, such as separation damage and thermal damage.^[17,18]^

Based on the situation above, we improved the methods of finding RLN and named it *lateral approach*. First, we broke the middle thyroid vein to expose the common carotid artery, then continued separating to the thymus levels in the caudal direction and assured to protect the thymus vein which was important for protecting IPG in situ. The lateral approach mainly relies on the conditions that the right recurrent laryngeal nerve intersects with the common carotid artery on the right side and the laryngeal recurrent nerve is close to the surface of the esophagus on the left side. In this case we can quickly find the laryngeal recurrent nerve in the lower part of the neck and separate along the surface of the RLN to the point where the RLN gets into the larynx close to the thyroid gland with the anatomical advantages including fewer blood vessels, avoiding bleeding, and loose connective tissue. Since the recurrent laryngeal nerve is attached to the underlying fibrous tissue during the separation, it is not continuously pulled and always in a low tension state, so the chance of physical damage is very low. Our experimental results also showed that the test group required a shorter operative time and a lower injury of recurrent laryngeal nerve mostly because of a significant reduction of intraoperative bleeding and series of operations caused by bleeding.

When the recurrent laryngeal nerve was separated to the laryngeal entry point, we continued to separate upward to deal with the upper parathyroid separation, and the operation was also required to be close to the thyroid gland. The key point of the operation mentioned above was to confirm that RLN had entered the larynx and protected the parathyroid blood supply.^[19]^ The former can avoid nerve damage for angle-forming nerve caused by the glandular tissue pull. The latter should be noticed that operation must be close to thyroid tissue as much as possible to preserve the blood supply of the upper PG.

Since the RLN in the neck has been exposed by the previous operations, it is safe for the RLN when the inferior PG separation is performed boldly at this time. As we know that the inferior PG is mainly pulled down by the thymus,^[20]^ so the inferior PG may appear in any pathway of thymus development process. In other words, it is closely related to the thymus gland in which we call it TRIPG. In our operation, it is easy to find thymus on the superficial surface of the common carotid artery in the lower part of the neck. Then on this basis, looking for the lower parathyroid tissue retrograde becomes easier. Generally we can find inferior PG in or near the top of the thymus, or in the fibrous connective tissue along the top of the thymus, or in the surface of the thyroid gland. If the blood supply is poor, inferior parathyroid will be transplanted in time in the muscle of the left forearm. It can be retained in situ if the blood supply is in good condition during the operation. In our study the inferior parathyroid recognition rate and the retention rate in situ of the test group were higher than those of the control group. Meanwhile the results of our postoperative review revealed that the PTH level in our study was also higher than that in the control group in the 1-day and 2-month follow-up. It may illustrate its feasibility of the notion called TRIPG in the fast recognition of parathyroid. But there is also a disadvantage in our practice that no methods are available for verifying the blood supply of the PG, which may be solved by the intraoperative validation with indolephilophilic green.^[21,22]^

It should be pointed out that our study has its limitations. Firstly the present study includes a relatively small number of patients even though some meaningful results have been obtained; secondly the utilization rate of IONM is fairly low for its high expenses (due to China's healthcare policy), intraoperative nerve function is difficult to be guaranteed without IONM; thirdly indolephilophilic green technology has been not used for verifying the blood supply of the PG. Besides, the TRIPG is an empirical summary of our single center which requires to be verified by more medical centers in the future.

Based on the clinic practice mentioned above, we believe that the improved method of finding RLN is simple and feasible with lower risk of damaging important organs and less difficulty in surgery especially in hospitals without intraoperative neuromonitoring equipment, or for the male patient with short and thick neck.

## 5 Conclusion

An improved method of finding the recurrent laryngeal nerve is a simple, efficient and safe way, easy to implement. Meantime it is beneficial for the surgeons to achieve a better clinical effect by finding RLN and protecting PG in a more efficient way.

## Author contributions

**Conceptualization:** Wenxin Zhao.

**Data curation:** Chao Xie.

**Investigation:** Liyong Zhang.

**Methodology:** Wenxin Zhao

**Project administration:** Shouyi yan.

**Resources:** Shouyi yan.

**Software:** Bo Wang.

**Writing – original draft:** Shouyi Yan.

**Writing – review & editing:** Shouyi Yan.
